# Chelmsford—Charge of Murder—Acquittal on the Ground of Puerperal Insanity

**Published:** 1848-07-01

**Authors:** 


### CHELMSFORD—FRIDAY, March 10.

### CHARGE OF MURDER.—ACQUITTAL ON THE GROUND OF PUERPERAL INSANITY.

(*Before Lord Denman.*)

MARTHA PRIOR, aged 37, a married women, was indicted for the wilful murder of her female child, 13 days old, by nearly cutting off its head with a razor.

Mr. RYLAND prosecuted. The prisoner was defended by Mr. HAWKINS.

It appeared from the evidence of the witnesses for the prosecution, that the prisoner is the wife of a labouring man, and resided at a village called North End, in the parish of Great Waltham, in Essex. She was pregnant in December last; and it would seem that she had made the usual preparations for her confinement, and was delivered on the 10th of December of the child which formed the subject of the present inquiry. The prisoner went on very well for some days after her confinement; but from symptoms which the medical attendant then observed, he gave directions that she should be kept perfectly quiet, and that the child should not be allowed to go to her, and also that the prisoner should not be left by herself. In consequence of these directions, it appeared that for two or three days the prisoner was attended and watched by two women belonging to the parish; but on the 23rd of December, the day when the alleged murder was perpetrated, it seemed that they had left her, and there was no one in the cottage but the infant and a daughter of the prisoner's, named Ellen, about 13 years old, who had the care of it. About one o'clock in the day, the prisoner, who was in bed, called to the girl Ellen, and told her to bring her the child. The girl at first objected to do so, but the prisoner, in an angry and peremptory tone, insisted that she should, and the girl being alarmed, carried the child to her mother, and laid it on the bed by her side. A short time afterwards, the prisoner called out again to her daughter, and told her to bring her a razor. She asked her what she wanted with a razor, upon which the prisoner said she wished to cut some of the hard skin off her hands. The girl, fearing some mischief, remonstrated with her mother, and asked her not to have the razor till another time; but she again became very angry; and the girl, it appeared, being terrified, took her one of her father's razors; but the moment she had done so, ran out of the cottage, intending to give an alarm, but she had hardly reached the door before she heard the infant give a shriek, and on her going into her mother's bedroom, accompanied by some of the neighbours, immediately afterwards, it was discovered that the child was dead, its head having been nearly cut from the trunk, and only remaining attached to it by some portion of the integuments. The prisoner appeared quite calm and collected, and she at once admitted that she had destroyed the child, and said it was what she had all along intended to do; and she added, that she should not care if any one served her the same. The women who had watched with the deceased during the first period of her illness, said that she frequently seemed as though her mind was roving, and often said that she knew she was going to die, and, as she was certain she should go to hell, it might be as well first as last, and other incoherent expressions. All the witnesses concurred in giving the prisoner the character of a kind and affectionate mother up to the period of this unfortunate transaction.

Mr. Bell, a surgeon of Felstead, proved the circumstances connected with the delivery, and said, that when he left the prisoner after it took place, she was doing very well. He was called in to see her about a week after, and he found her in a dangerous state, suffering under a complete prostration of strength. Her eyes were vacant and wild, and her countenance haggard; and from what he saw, he was satisfied that her mind was affected, and he in consequence gave directions that the child should not be given to her, and that she should be kept perfectly quiet, and not be permitted to be alone. The witness concluded his evidence by expressing an opinion that at the time the prisoner *destroyed the child, she was not aware what she was doing, or if she was, that she was incapable of controlling her actions.*

Lord Denman asked the witness whether he was of opinion that when the prisoner sent for the child she was not aware what she was about?

The witness said, that it was his opinion that the prisoner might have known that she was going to kill the child, but at the same time was not aware of the nature of the act she was about to commit; in point of fact, that she acted under a sudden and uncontrollable impulse of the mind.

Lord Denman.—Do you call it a sudden impulse of the mind when a person deliberately asks for a child, and then suffers a quarter of an hour to elapse before the razor is asked for with which the injury then appears to be deliberately inflicted?

Witness.—I am of opinion that she committed the act under an uncontrollable impulse acting upon a mind previously diseased.

Lord Denman then addressed the jury, and said, that after this evidence, he had no doubt they would come to the conclusion which it was intended to convey, that the prisoner, although she had no doubt committed the act imputed to her, was not legally responsible for it. He must, however, express an opinion that the judgment of the medical gentleman *had been very rashly formed.* How could one person dive into the mind of another, and express an opinion with regard to its being in an unsound state, when there was no evidence of any alteration of conduct, or any circumstances in the case to show alienation of mind? He said this on account of the great danger that would prevail to human life if people were to be taught that a sudden impulse was to be an excuse for a crime, and that the atrocity of the offence itself was to be adduced as an argument in support of such a supposition. He could not help thinking that such opinions were too often given by scientific men upon too slight foundation for the safety of the public; but as he felt that in this instance there *was no doubt that the jury would act upon the testimony of the medical gentleman* who had been examined, it would be useless to proceed any further with the inquiry.

The jury accordingly returned a verdict of *Not Guilty*, on the ground of insanity.

